# Home-Delivered Nutrition Services for Older Adults Under the Older Americans Act

**DOI:** 10.1001/jamanetworkopen.2025.34747

**Published:** 2025-09-30

**Authors:** Em Balkan, Emily A. Gadbois, Emma L. Tucher, Kimberly P. Bernard, Kali S. Thomas

**Affiliations:** 1 Department of Health Services, Policy and Practice, Brown University School of Public Health, Providence, Rhode Island; 2 Center for Gerontology & Healthcare Research, Brown University School of Public Health, Providence, Rhode Island; 3 Kaiser Permanente Northern California Division of Research, Pleasanton; 4 Survey, Qualitative and Applied Data Research Core, Brown University School of Public Health, Providence, Rhode Island; 5 Center for Equity in Aging, Johns Hopkins School of Nursing, Baltimore, Maryland

## Abstract

**Importance:**

The Older Americans Act Nutrition Program is essential to supporting older adults and their ability to maintain their quality of life. Despite this, such services are underfunded and are at risk of further cuts.

**Objective:**

To characterize the benefits of home-delivered meals programs as reported by older adult recipients.

**Design, Setting, and Participants:**

This qualitative study is a substudy within a randomized clinical trial, Deliver-EE, which evaluates the comparative effectiveness of 2 modes of meal delivery. Participants were randomized to 1 of 2 study groups: daily-delivered meals or frozen, bulk-shipped meals. Between August 2022 and March 2024, in-depth, semistructured telephone interviews with older adults receiving home-delivered meals were conducted. Interviews were conducted with participants living in California, Florida, North Carolina, South Carolina, and Texas. The interview guide was designed in collaboration with 2 advisory panels. The guide covered the quality and impact of meals.

**Exposure:**

Receipt of home-delivered meals.

**Main Outcomes and Measures:**

The primary outcome was to examine the benefits of home-delivered meals programs. Interview transcripts were analyzed using modified grounded theory and thematic analysis.

**Results:**

According to interviews with 54 participants (mean [SD] age, 75.9 [6.8] years; 30 female [55.6%]), home-delivered meals met the stated goals of the Older Americans Act Nutrition Program (to reduce hunger, food insecurity, and malnutrition; to promote socialization; and to promote the health and well-being of older adults). Participants said that home-delivered meals provided much-needed nutritional stability and that they had positive interactions with delivery drivers. Meals also relieved their stress, including by eliminating their need to cook for themselves, which can be physically taxing. In addition, participants said home-delivered meals improved the health, well-being, and finances of their caregivers.

**Conclusions and Relevance:**

The findings of this qualitative study of home-delivered nutrition services for older adults suggest that home-delivered meals programs achieved their intended outcomes and yielded meaningful benefits beyond their stated purpose that remain to be quantified. The findings also support funding the Older Americans Act Nutrition Program.

## Key Points

**Question:**

What are the benefits of the Older Americans Act’s home-delivered nutrition services to older adult recipients?

**Findings:**

This qualitative study of 54 participants found that home-delivered meals meet the Older Americans Act’s stated goals to (1) reduce hunger, food insecurity, and malnutrition; (2) promote socialization; and (3) promote the health and well-being of older adults. In addition, participants said home-delivered meals improved the health, well-being, and finances of their caregivers.

**Meaning:**

These findings suggest that home-delivered meals programs not only achieve their intended outcomes but also yield meaningful health-related benefits beyond their stated purpose that remain to be quantified.

## Teaser

This qualitative substudy of the Deliver-EE randomized clinical trial assesses whether 2 modes of meal delivery to older adult recipients meet the goals of the Older Americans Act Nutrition Program, including reduction of hunger, food insecurity, and malnutrition and promoting socialization and well-being.

## Introduction

It is well understood that older adults benefit from nutritional support. As such, the federal government supports the provision of food to older adults through the Older Americans Act (OAA) Nutrition Program.^1^ In 1972, the OAA was amended to include a Nutrition Program^1^ with 3 objectives: (1) reduce hunger, food insecurity, and malnutrition; (2) promote socialization; and (3) promote the health and well-being of older adults.^2^ Funding levels for the OAA Nutrition Program are set by Congress during annual appropriations processes.^3^ These funds are used by state and local aging agencies to administer OAA nutrition services primarily through contracts with nonprofit community-based organizations.^3,4^ This includes Meals on Wheels (MOW) organizations.^3^ According to MOW America, OAA’s Title III C funding covers approximately one-third of the costs in providing 251 million meals to 2.2 million homebound older adults each year, making this an integral source of funds for home-delivered meal programs.^5,6^

There are substantial unmet nutritional needs for older adults despite funding of home-delivered meals programs. According to a 2024 report by the US Government Accountability Office,^7^ 86% of older adults who have low incomes and experience food insecurity do not receive home-delivered meals. Meanwhile, home-delivered meal recipients report very high satisfaction with services.^8,9,10^ Although qualitative and quantitative research suggests these programs provide nutritional benefits, improve recipients’ health and well-being, and reduce health care utilization,^9,11,12,13,14^ the most recent national evaluation of the OAA Nutrition Program using survey data found no differences in food insecurity and social isolation between participants and nonparticipants who had low incomes.^10^ The study noted limitations, including that the nonparticipants were a matched sample of Medicare participants not receiving meals, which could introduce bias.^10^ Given these findings, previous questions about the state of evidence for home-delivered meals programs,^15^ and recent threats to program funding,^16^ the objective of this study is to continue to examine the benefits of home-delivered meals from the perspectives of participants in a large, national randomized clinical trial of home-delivered meals.

## Methods

### Study Design

This is a qualitative substudy within a randomized clinical trial, Deliver-EE, which is being conducted with 2300 older adults on waiting lists at 13 MOW programs across the US.^17,18,19^ Once recruited to the study, participants are randomized to receive 1 of 2 modes of meal delivery: hot or chilled meals delivered 5 days a week by a paid or volunteer driver, or meals that are frozen and mailed to participants’ homes every other week in bulk. During the period of our qualitative substudy, we sought to interview 8 individuals from each of the 7 MOW sites actively enrolling participants to achieve saturation^20^ using purposive sampling. Our study was designed to assess perceptions of meal delivery programs for individuals in both groups of the study, stratified by living arrangement (living alone vs not living alone) and preferred meal type at baseline. From each site, we sought to recruit 4 individuals from each study group, 2 of whom lived alone and 2 who did not live alone. Of those 2, 1 received their preferred meal at baseline whereas the other did not receive their preference. We recruited participants representing diversity in race and ethnicity, and we required participants to have received meals for at least 3 weeks before our interviews.

We recruited individuals for telephone interviews using an approved recruitment script. This study, including recruitment and interview materials, was approved by Advarra’s institutional review board. Advarra is an independent organization fully accredited by the Association for the Accreditation of Human Research Protection Programs.^21^

This study followed the Consolidated Criteria for Reporting Qualitative Research (COREQ) reporting guidelines. Researchers, which included faculty members, staff, and students, were compensated for their time, identified as women or nonbinary, and had research experience ranging from 2 to 10 or more years.

### Interview Protocol

The research team designed an interview guide in collaboration with the study’s 2 advisory panels.^17^ Although largely consistent, there were selected relevant questions tailored to each mode of food delivery within the guide (see the eAppendix in Supplement 1). After conducting 3 pilot interviews with current meal recipients at our participating sites, the interview protocol was revised for clarity.

### Interview Procedures

Interviews were conducted between August 2022 and March 2024 by 4 coauthors (E.B., E.A.G., E.T., and K.P.B.). One team member conducted the interview while another took notes. Interviews were conducted over the telephone and recorded, with participants’ verbal consent obtained at the beginning of each interview. Participants were told the purpose of the interview, which was to learn about their experiences to improve home-delivered meals programs and understand their impact. Researchers clarified that they are not employed by meal providers. Researchers had no prior relationship with participants. Interviews lasted approximately 30 to 50 minutes. Participants were in locations convenient and private for them. Interviewers were in their work offices. Interviews were transcribed by a professional transcription company, then reviewed, corrected (as needed), and deidentified. Each participant was mailed a gift card valued at US $50 for participation. No interviews were repeated, and transcripts were not provided to interviewees.

### Statistical Analysis

We analyzed the interview transcripts using modified grounded theory and thematic analysis.^22,23^ We created a preliminary coding scheme based on the questions asked during the interviews (inductive) and from the study team’s interpretations of what was being heard in interviews (deductive) using axial coding. The coding scheme was refined collaboratively after testing it by coding 2 transcripts. Transcripts were double-coded using Microsoft Word, with 4 researchers independently coding transcripts and meeting in pairs on a weekly basis to resolve coding disparities and discuss overall themes. Weekly team meetings were held to discuss emerging themes, reconcile coding differences, and ensure coding rigor. Coded data were entered into the qualitative software package NVivo R1.7 (QSR International).

Once all interview transcripts were coded, reports were generated in NVivo. Using thematic analysis, selected coding reports were reviewed and an overarching theme for the manuscript was outlined using OAA Nutrition Program’s goals. The first theme addresses the first goal of the OAA Nutrition Program: to reduce hunger, food insecurity,^24^ and malnutrition.^25^ The second focused on socialization, and the third on the promotion of health and well-being.^26^ Findings were discussed with both advisory panels to seek clarification and insights, as a form of member checking.

## Results

Of the 90 participants we contacted, 36 (40%) declined to participate in the interviews. Of those who declined, 26 (72%) passively declined (eg, unreturned voicemails), 7 (19%) declined because they did not want to be interviewed or could not be because of language barriers, and 3 (8%) declined for health reasons (eg, were hospitalized). Interviews were conducted with 54 participants (mean [SD] age, 75.9 [6.8] years; 30 female [55.6%]) from 7 sites. Participants lived in California, Florida, North Carolina, South Carolina, and Texas. Participants varied racially, ethnically, in marital status, and need for assistance^27^ (Table 1).

**Table 1.** Baseline Characteristics of Participants

Characteristic	Participants, No. (%) (N = 54)
Self-reported race	
Asian	1 (1.9)
Black	15 (27.8)
White	31 (57.4)
Multiple	3 (5.6)
Other^a^	4 (7.4)
Age, y	
Mean (SD)	75.9 (6.8)
Age categories	
66-70	14 (25.9)
71-75	13 (24.1)
76-80	11 (20.4)
81-85	10 (18.5)
86-90	6 (11.1)
Sex	
Female	30 (55.6)
Male	24 (44.4)
Lives alone	27 (50.0)
Needs assistance	
With activities of daily living^b^	20 (37.0)
With instrumental activities of daily living^b^	40 (74.1)

^a^ Participants were asked to describe their race, and 4 self-identified as a race other than Asian, Black, White, or multiracial.

^b^ Our baseline survey included questions about assistance with personal care and routine needs, such as “Because of any impairment or health problem, do you need the help of other persons with your personal care needs, such as eating, bathing, dressing, or getting around the house?” We reported these responses as assistance with activities of daily living (ADLs). Other questions included, “Because of any impairment or health problem, do you need the help of other persons in handling your routine needs, such as household chores, doing necessary business, shopping, or getting around for other purposes?” We reported these responses as instrumental activities of daily living (IADLs). Thus, individuals who responded affirmatively had 1 or more ADLs or IADLs. See the definitions of ADLs and IADLs published elsewhere.^27^

Our analysis of the interviews revealed 4 themes, described in the following subsections. We include representative quotations for the themes and key concepts across sites and intervention groups in the tables.

### Theme 1: Nutritional Benefits From Home-Delivered Meals, Including Reduced Hunger, Food Insecurity, and Malnutrition

Participants stated that prior to receiving meals, they relied on takeout or simple meals that often lacked nutrition. One participant said, “I don’t eat healthy if I fix my own food,” and that, aside from delivered meals, they eat “something that I can fix real quick without standing up” (participant 1). Furthermore, some participants shared that they previously were not sure where their next meal would come from, and they would experience hunger. One participant said that, “There [were] times when I would run [out of food], but not since I’ve been on Meals on Wheels” (participant 2). Many participants noted that receiving meals improved their financial security, which increased their access to nutritious foods. One participant said, “It changes…what I [choose] in the grocery store” including more “fresh fruit” (participant 3). One participant, who previously needed to get their groceries delivered, said, “It was hard for me to make my pennies go farther through the month. But that’s what I had to do cause a lot of times I was so ill, I didn’t even feel like getting out of the house” (participant 4). For representative quotations, see Table 2.

**Table 2.** Theme 1: Nutritional Benefits From Home-Delivered Meals, Including Reduced Hunger, Food Insecurity, and Malnutrition

Key concept	Representative quotation
Increases nutrient intake, specifically vegetables	“So I find that I’m eating more vegetables than I ever have in my life…. So to me this is wonderful having someone else making me a healthy meal. I really appreciate it.” (participant 13)
Addresses food insecurity and hunger	“I used to be hungry and now I’m not.” (participant 14)
Provides access to nutritionally balanced meals	“When I started getting these meals, I started eating a more well-balanced meal, because when I’m here by myself, there’s no food, there’s no vegetables, or just to grab a sandwich or something. So I have a more balanced meal with these meals.” (participant 15)
Improved nutrition for those who cannot prepare their own food	“I eat a lot of beans because you don’t have to see how to make beans…. But far as frying and mixing food, I can’t do that because of my sight. I haven’t learned to manage my sight yet.” (participant 16)
Meets the nutritional needs of those with chronic conditions	“Yeah, it help you with your diabetes too. It help keep your sugar down too because I’m not eating a whole lot of other things you shouldn’t be eating. And Meals on Wheels is more healthy for that. It probably help you lose some weight off of that too.” (participant 17)
Allows individuals to afford higher quality food, which addresses food insecurity	Participant: “Yeah, I get to be a little more picky at what I’m buying. Buy the better cuts of meat and the good vegetables.” Interviewer: “That’s awesome. And have you noticed any other changes since you started getting the meals?” Participant: “My blood glucose is stable, pretty good. I’m diabetic so I test every day.” (participant 18)
Allows food stamps to cover a higher proportion of individuals’ groceries	“I don’t have to worry whether the food stamps are going to make it until the end of the month, because usually they don’t make it until the end of the month…and maybe we don’t make as many trips to the grocery store too, because I don’t need as much either.” (participant 19)

### Theme 2: Positive Social Interactions With Delivery Persons

When we asked participants receiving daily-delivered meals about their interactions with delivery drivers, we heard, overwhelmingly, that the drivers were polite and pleasant. Participants expressed feelings of being well taken care of. One participant shared, “I told you about the drivers, and I think you picked the right people to do that job. They’re always really nice.... At first, I started to feel like I’m on the corner with the paper cup and all that. They don’t make you feel that way” (participant 5). During some interviews, we captured the interaction between the participant and delivery person. See Table 3 for one such example (participant 6).

**Table 3.** Theme 2: Positive Social Interactions With Delivery Persons

Key concept	Representative quotation
Drivers engage in polite and short conversation	Participant: “…Oh, there they are now, in fact. …. Hi, how are you? Thank you so much. I appreciate you. Thank you.” MOW driver: “You are more than welcome.” Participant: “Thank you so much.” MOW driver: “You seem to be doing a lot better.” Participant: “I am getting better. It’s a blessing to say I’m so happy to just be here.” MOW driver: “That’s because of the food.” Participant: “Yes. Yes. Thank you so much.” MOW driver: “You’re welcome. Have a wonderful day.” Participant: “You too. You be safe.” (participant 6)
Participants enjoy talking to the delivery person	“Yeah, it’s nice to sit and talk to them for a minute or so when they come up and everything. They’re very sociable people, and I think it’s real nice that they’re there.” (participant 21)
Participants are concerned about keeping drivers from other deliveries	Participant: “We chat for a minute, a half a minute or two. ‘How your day going? What’s happening?’ They’re really pleasant people, really concerned about what’s going on.” Interviewer: “Well, that’s nice. So we know from some people that sometimes the drivers might help clients with things like bringing in the mail or changing a light bulb or something.” Participant: “Oh no, no, No. That never happens. No, I wouldn’t expect for them to even do stuff like that because I know they got a delivery to make, and sometime I even open up my door and go out there before they even get here…and I thank them for bringing it[.]” (participant 27)
Participants share that meal delivery drivers would help them if needed	“I’m fortunate where I do have friends in the building. So personally, I would feel funny asking somebody to help me get something, even help something off the shelf. Unless it arose…they happened to be there, yes, I wouldn’t mind asking. And I’m sure that they would bend over backward to help you.” (participant 7)

Abbreviation: MOW, Meals on Wheels.

Many participants shared that they did not want to bother drivers with conversation because they thought the drivers needed to rush to get to other deliveries. However, participants reported being confident drivers would “bend over backwards” (participant 7) to assist them if they asked for help. In fact, a few individuals stated that the drivers had assisted them. One participant said, “I have asked them to buy me some stamps from the post office…. He always brings them the next day” (participant 4). That same participant shared that, “It gives me a chance to talk to somebody. I live here alone, so that helps being able to converse with somebody every day.” For representative quotations, see Table 3.

### Theme 3: Improved Well-Being, Including Improved Mood and Physical Health

Some participants expressed that receiving meals improved their well-being. Participants said that their health, as well as their mood, improved because of the nutritional quality of meals. One participant shared, “I feel better because I’m eating wholesome food instead of running, getting [fried] chicken…or something like that” (participant 8).

Many participants said that the meals improved their well-being by reducing stress. Participants with chronic conditions expressed relief at being able to rely on delivered meals. During an interview, one participant stated:

It’s a lifesaver for me because…I’m to the point with [chronic obstructive pulmonary disease] now that I cannot cook and clean the kitchen up in the same day. I have to cook and then rinse everything out and set it in the sink, and do the dishes the next morning…. I just don’t have enough air to cook it and clean up in the same day (participant 9).

Participants expressed appreciation for the ease and convenience of the program, with one participant stating, “I was trying to gain weight…. So it was convenient for me to have something already made up because I wasn’t in the mood for food that much” (participant 10).

Participants also said that they think meals can improve well-being by helping people maintain autonomy, which can also help them stay in their homes. One participant shared, “I live by myself and when I fell back in July, I bruised my kidney and this took me a while to get over it and I was debating about having to go to a nursing home and thank goodness I didn’t have to. And so these meals and all helped me to stay in my own home” (participant 11). For representative quotations, see Table 4.

**Table 4.** Theme 3: Improved Wellbeing, Including Improved Mood and Physical Health

Key concept	Representative quotation
Meals relieve stress	“I’m probably a little less stressed, and maybe a little more relaxed. Not knowing that, ‘Oh geez, I got to cook.’” (participant 22)
Meals help those with physical challenges, increasing well-being	“It makes it a lot easier on me…I don’t have to stand in the kitchen for 45 min to an hour. Because that’s my big thing is standing with very little balance. I don’t have to sit there and make a whole hour project.” (participant 23)
Meals assist those managing chronic health conditions and help people feel better	“Now, I am eating better, more healthy…. I feel better because there is something that I don’t have to be worry about, especially when I have my appointments and everything…today I have one this afternoon…. I have my lunch and then I can go and do what I have to do…it’s helping me a lot. I appreciate it a lot.” (participant 24)
Meals help people avoid going to the hospital or nursing home	Interviewer: “Do you think that the meals, the Meals on Wheels can help people avoid going to the hospital or a nursing home….” Participant: “I think so. I think it helps…if they’re afraid to cook or if they don’t have the money to afford all the groceries they need. And that’s one thing that will benefit…especially if they’re home bound or they don’t get out very much…. It helps them be able to get the food that they need to survive. Especially if they have on their own fixed income, they got medications they got to buy or utilities or whatever. And then if they don’t have a lot of family that can help them or neighbors that they don’t see very often....” (participant 4)

### Theme 4: Improvements of the Well-Being and Finances of Caregivers

Many participants expressed relief at not needing to rely on others for support getting groceries. One participant said, “It’ll help you out far as you get hungry and you waiting on somebody to take you to the store or go to the store for you. So you have something right there for you. And you don’t have to worry about going anywhere” (participant 12). Other participants shared that their caregivers seemed relieved by the program, allowing them to spend less resources caring for their loved one. One participant said:

I have [my sisters] close by and they were having to come for dinner and supper to make sure I could fix me something…. And I’m so glad that I’ve got that so they don’t have to worry about me having something to eat all the time.... [I think] they’re feeling a lot relieved (participant 1).

We also spoke with participants who themselves were caregivers to spouses, children, grandchildren, and other individuals. These participants noted that receiving meals allowed them more time to relax and spend quality time with family. One participant said, “We don’t always have everything for [my brother] to have three meals a day. He’s more of a finger food kind of guy, so I try to make sure there’s hot dogs and stuff like that. But to have at least one square meal a day makes a big difference for him. It’s a nice break for me because I don’t have to worry about it. I know that at least for one meal a day, he’s got everything he needs” (participant 5). For representative quotations, see Table 5.

**Table 5.** Theme 4: Improvements of the Wellbeing and Finances of Caregivers

Key concept	Representative quotation
Caregivers of participants are relieved and less stressed because of meals	“Now I feel like I don’t have to worry about the meals getting low, because I always have enough now…. [It’s] not my son’s fault [meals got low], because he don’t know. And he wastes so much money because of my appetite, and he always tell me to let him know [what I need,] because if he just up and bring things here, I can’t eat it because my appetite just won’t let me…. They want me to move with them, but I don’t want to. I want to keep my independence as long as I can.” (participant 2)
Participants express relief owing to relying less on others	“It’s a godsend to me. I don’t have to bother somebody to take me to the store, and then they have to come in and shop with me because of my eye condition. And it’s a nuisance to somebody…. Everybody has problems. I don’t want them burdening me with their problems, so I try not to do that.” (participant 25)
Participants report improved feelings of independence by no longer needing to rely on caregivers	“Well, I know people that that’s probably why they end up in nursing homes or have to have somebody come in and care for them because they’re not getting a good nutrition. I think it’s going to impact, my wife and I get to stay in our home a lot longer. I’ll be 80 in January and I want to be here as long as I can.” (participant 18)
Participants who are caregivers report improved well-being	“Well, I’m the one responsible for the meals and I don’t like having to cook because I’m a messy cook…. I have three spoiled dogs and a semi-invalid wife, and it just makes things a lot easier around here.” (participant 26)

## Discussion

In this qualitative study, participants reported that home-delivered meals met—and even surpassed—the 3 intended purposes of the OAA Nutrition Program: to reduce hunger, food insecurity and malnutrition; to promote socialization; and to promote health and well-being.^2^ An important finding of our study is that home-delivered meals support caregivers. More than 1 in 5 individuals in the US provides unpaid care for a family member, and, of these, nearly 80% do so for an adult older than 50 years.^28^ We documented how meals relieved stress related to caregiving, including by providing caregivers with more time to care for themselves. These findings align with previous interviews with caregivers of home-delivered meal participants^29^ and advance the evidence base around the ability of home-delivered meals to impact recipients’ broader social and care communities. Future research should continue to examine the spillover effects of these programs.

Despite the growing body of evidence documenting the benefits of home-delivered meals, including those presented here, federal funding for the program has grown only incrementally and not kept pace with the growth in the aging population or inflation.^30^ In fact, if total OAA appropriations had kept up with inflation since fiscal year 2001, funding for fiscal year 2024 would have been $620 million more than actual funding just to maintain the same total buying power over time.^30^ Given the increase in the aging population, rising food costs, and inadequate support for older adults in their communities, there is an urgent need to address the substantial unmet need for nutrition services among older Americans. This study suggests that the OAA Nutrition Program delivers on its goals.

The holistic benefits of home-delivered meals—some of which have yet to be quantified—should be taken into consideration as policymakers decide on the continued funding of the OAA. In their own words, participants were healthier, more financially secure, and happier because of this program. We can infer that cuts to the program would result in increased food insecurity, financial strain, and health complications. Importantly, the vast majority (86%) of older adults who have low incomes and experience food insecurity do not receive home-delivered meals, so these positive benefits are only experienced by a fraction of those who are in need.^7^ Our findings suggest the program is meeting its intended goals—in addition to supporting caregivers, a national priority as reflected in the RAISE Family Caregivers Act, which became law in 2018^31^—and warrants additional resources to meet the growing needs of this population.

Our findings are also relevant to health insurers, including Medicare Advantage plans that offer supplemental benefits such as home-delivered meals,^32^ and Medicaid programs that serve older adults. MOW America reports that 1 year of home-delivered meals costs approximately the same as a single day in the hospital or 10 days in a nursing home,^33^ and previous research found that home-delivered meals help keep older adults with low-care needs in the community and out of nursing homes.^12^ Health care entities could benefit from further supporting home-delivered meals programs and by connecting beneficiaries to such programs.

### Limitations

Our findings should be interpreted in light of their limitations. First, when examining socialization as a benefit (theme 2), we chose to only include daily-delivered meal recipients. We do not expect individuals who receive frozen meals delivered by postal couriers every 2 weeks to have substantive social interactions with the people delivering their meals. Second, all interviews were conducted 3 to 6 months after the initial meal delivery. It is possible that perceptions about meals continued to change and evolve over time. Third, all participants were recruited from programs with waiting lists for meal services. Meal programs with active waiting lists may be different from programs without waiting lists in terms of available resources. Fourth, although study participants speak both English and Spanish, qualitative interviews were only able to be conducted in English. Future qualitative research is needed to understand how meals meet the needs of non-English speakers.

## Conclusions

In this qualitative study of home-delivered nutrition services for older adults, we found that home-delivered meals programs supported by the OAA provided numerous benefits to older adults, their caregivers, and their communities, which far surpassed their intended impacts. Despite this, current federal funding is not sufficient to meet the needs of all older adults who could benefit from these services^7,30^ and recent threats to the program^15,16^ have the potential to greatly harm older adults who rely on this service. The findings of our study suggest that home-delivered meals programs provided a substantial return on investment that remains to be fully quantified. These findings further justify increased funding for these programs. As the US population ages and food insecurity increases, the funding provided by the OAA Nutrition Program is essential to supporting home-delivered meals programs and the many older adults who rely on this service.

## Notes

Supplement 1. eAppendix. Overview of Interview Guide

Supplement 2. Data Sharing Statement
