# Supplementary material for: Home-Delivered Nutrition Services for Older Adults Under the Older Americans Act
Source: JAMA Netw Open. 2025 Sep 30;8(9):e2534747. doi: 10.1001/jamanetworkopen.2025.34747 (PMC12485633; doi:10.1001/jamanetworkopen.2025.34747)
Supplement: Supplement 1. — eAppendix. Overview of Interview Guide [file jamanetwopen-e2534747-s001.pdf]

Supplemental Online Content

Balkan E, Gadbois EA, Tucher E, Bernard KP, Thomas KS. Home-delivered nutrition services for older adults under the Older Americans Act. *JAMA Netw Open*. 2025;8(10):e2534747. doi:10.1001/jamanetworkopen.2025.34747

eAppendix. Overview of Interview Guide

This supplemental material has been provided by the authors to give readers additional information about their work.

© 2025 Balkan E et al. *JAMA Network Open*.

eAppendix. Overview of Interview Guide

Introduction
Before we get started, I'd like to know how you'd prefer for me to address you during the interview. What should we call you?

Next, I want to say again, thanks so much for talking with me! During today's interview we're hoping to hear about how it has been to receive meals so that the program can improve.

This interview is entirely voluntary and confidential. You can end the interview at any time and can decline to answer any question you'd like. Because we're asking about your experiences and perspectives, there are no right or wrong answers. For today's interview, you'll receive a $50 gift card for taking part in the interview.

I'd like to record this interview and hope this is okay with you - do I have your permission to record it?

Do you have any questions for us before we begin?

Interview Questions

1. First, are you still receiving meals?

    If you are no longer receiving meals, we'd still like to talk with you.

    - Please tell us why you decided to stop getting meals at your home.
    - Is there anything that the program could have changed that would have made you want to keep getting meals?

2. We would like to know what it's like to get meals at your home. Do you receive daily delivered meals or drop-shipped frozen meals?

3. Questions for those with **daily delivered meals**:
    a. What usually happens? How does the meal get inside your home?
        i. Does anyone help you bring in the meal or set it up?
    b. When do the meals usually come? How do you know when they're coming?
    c. Some people say it's hard not knowing when meals are coming; some say that waiting for the meals throws off their day. How has this been for you?
    d. Now let's talk about the person who brings the meals.
        i. Do you talk with the person? Do you look forward to seeing them?
        ii. If there are more than one person, is there one you like more than another?
        iii. What else can you tell us?

© 2025 Balkan E et al. *JAMA Network Open*.

e. Some drivers help clients in different ways, such as small chores like changing light bulbs, bringing in mail or a package. What about your experience?
    i. What kinds of things would you like drivers to help with, if they could?
f. About how much food would you say you get? About how much do you usually eat? Is there enough food? If you have leftovers, what do you usually do with them?
    i. Do you ever share the meal with anyone?
    ii. Do you ever share the meal with a pet?
    iii. Are there ever times when you have to stretch the meals out to make them last?
g. Talk about what you like about the food.
    i. Are there any unique, traditional, regional, or cultural foods you like to eat? Foods you used to cook for yourself at home? How do the meals you get now fit with those preferences?
h. This is very helpful. Again, there are no right or wrong answers. What you say helps us know how people feel about the meals and helps us improve them. Based on what we have discussed so far, what are your ideas about how the program might improve?
i. Overall, how much do you like getting meals delivered to your home? What do you like? What don't you like? What would you suggest be changed?

4. These questions are about **frozen meals**.
    a. What usually happens? How does the meal get inside your home?
        i. Does anyone help you bring in the meal or set it up?
    b. When do the meals usually come? How do you know when they're coming?
    c. Some people say it's hard not knowing when meals are coming; some say that waiting for the meals throws off their day. How has this been for you?
    d. Now let's talk about the person who delivers the meals.
        i. Do you talk with the person? Do you look forward to seeing them?
        ii. If there are more than one person, is there one you like more than another?
        iii. What else can you tell us?
    e. Tell us what it is like to store the frozen meals. Do you have enough space?
    f. What is it like to defrost and then prepare the frozen meals?
    g. Tell us about any challenges with storing or preparing the meals (storage, weight, working the microwave, other)
    h. About how much food would you say you get? About how much do you usually eat? Is there enough food? If you have leftovers, what do you usually do with them?
        i. Do you ever share the meal with anyone?
        ii. Do you ever share the meal with a pet?
        iii. Are there ever times when you have to stretch the meals out to make them last?
    i. Talk about what you like about the food.
        i. Are there any unique, traditional, regional, or cultural foods you like to eat? Foods you used to cook for yourself at home? How do the meals you get now fit with those preferences?
    j. This is very helpful. Again, there are no right or wrong answers. What you say helps us know how people feel about the meals and helps us improve them. Based on what we have discussed so far, what are your ideas about how the program might improve?

© 2025 Balkan E et al. *JAMA Network Open*.

k. Overall, how much do you like getting meals delivered to your home? What do you like? What don't you like? What would you suggest be changed?

5. What happens when you don't get meals, like on weekends? How do you get your food on those days?

6. Now think back to how it was before meals came to your home. How was that?

a. After you started receiving the meals, did you notice any changes in terms of your health? How about your mood?

b. How would you say getting meals has changed what you do during your days? For example, maybe now you don't have to make as many trips to the grocery store? At the grocery store, what has changed? How has it impacted what you buy? Or how much you have to spend? Or now that you have to wait for the delivery every day at lunch time?

c. We have heard from other people that getting these meals has had an impact on them financially. Have you experienced a financial impact from receiving meals?

7. Do you think meals can help people to avoid going to the hospital or a nursing home? Why might this be the case?

8. If you thought they needed them, would you suggest a friend or relative get these meals, too?

9. Now, finally, please tell us about other services you get to help you live at home.
   a. Do you get help from anyone else, like family members, friends, or neighbors?
   b. How does getting meals delivered fit with the other help you get?

10. What else should we know about getting meals that I have not already asked you about?

<u>Closing</u>

Thank you so much for speaking with us -- What you've had to say helps us know how people feel about the meals and how we can make this experience better for people.

As I mentioned at the very beginning, we will send you a $50 gift card for today's interview. What kind of gift card would you like?

© 2025 Balkan E et al. *JAMA Network Open*.
