# Supplementary material for: Home-Delivered Nutrition Services for Older Adults Under the Older Americans Act
Source: JAMA Netw Open. 2025 Sep 30;8(9):e2534747. doi: 10.1001/jamanetworkopen.2025.34747 (PMC12485633; doi:10.1001/jamanetworkopen.2025.34747)
Supplement: Supplement 2. — Data Sharing Statement [file jamanetwopen-e2534747-s002.pdf]

Data Sharing Statement

Balkan. Home-Delivered Nutrition Services for Older Adults Under the Older Americans Act. *JAMA Netw Open*. Published October 01, 2025. doi:10.1001/jamanetworkopen.2025.34747

Data
Data available: No

Additional Information
Explanation for why data not available: This is a qualitative study, and we cannot share our data.
